# Feasibility and safety of minimally invasive calcaneal osteotomy (MICO) through a medial approach: a case-control study

**DOI:** 10.1007/s00132-023-04460-9

**Published:** 2023-12-11

**Authors:** S. Beischl, N. Harrasser, A. Toepfer, C. Scheele, R. Smits Sererna, M. Walther, F. Lenze, H. Hörterer

**Affiliations:** 1 Clinic of Orthopaedics, Klinikum rechts der Isar, Technical University Munich, Ismaningerstr. 22, 81675 Munich, Germany; 2 Orthopaedics and Traumatology, Kantonsspital St. Gallen, Rorschacher Straße 95, 9007 St. Gallen, Switzerland; 3 Center for Foot and Ankle Surgery, Schön Clinic Munich Harlaching – FIFA Medical Centre, Harlachinger Straße 51, 81547 Munich, Germany; 4 Department of Orthopaedics and Trauma Surgery, Musculoskeletal University Center Munich (MUM), University Hospital, LMU Munich, Marchioninistraße 15, 81377 Munich, Germany

**Keywords:** Calcaneus, Minimally invasive surgical procedures, Flatfoot, Cavus foot, Kalkaneus, Minimal-invasive Chirurgie, Plattfuß, Hohlfuß

## Abstract

**Introduction:**

Minimally invasive calcaneal osteotomy (MICO) is already an established surgical procedure for correcting hindfoot deformities using a lateral approach. So far, no description of a medial approach for MICO has been published.

**Material and methods:**

Between August 2022 and March 2023, 32 consecutive patients (MICO with medial approach, MMICO: *n* = 15; MICO with lateral approach, LMICO: *n* = 17) underwent MICO as part of complex reconstructive surgery of the foot and ankle with concomitant procedures. The amount of correction in the axial view of the calcaneus and consolidation rates were evaluated radiographically. Subjective satisfaction, stiffness of the subtalar joint, and pain level (numeric rating scale, NRS) at the level of the heel were assessed clinically. The last follow-up was at 6 months.

**Results:**

All osteotomies consolidated within 6 months after surgery. Displacement of the tuber was 9 mm on average in either group. Relevant subtalar joint stiffness was detected in 5 MMICO and 6 LMICO patients. No relevant differences between the groups were detected for wound healing problems, nerve damage, heel pain or patient satisfaction.

**Conclusion:**

In this study lateral and medial approaches for MICO were performed. Similar degrees of correction and low complication rates were found in both groups. The medial approach for MICO is safe and can be beneficial regarding patient positioning and arrangement of the C‑arm.

**Graphic abstract:**

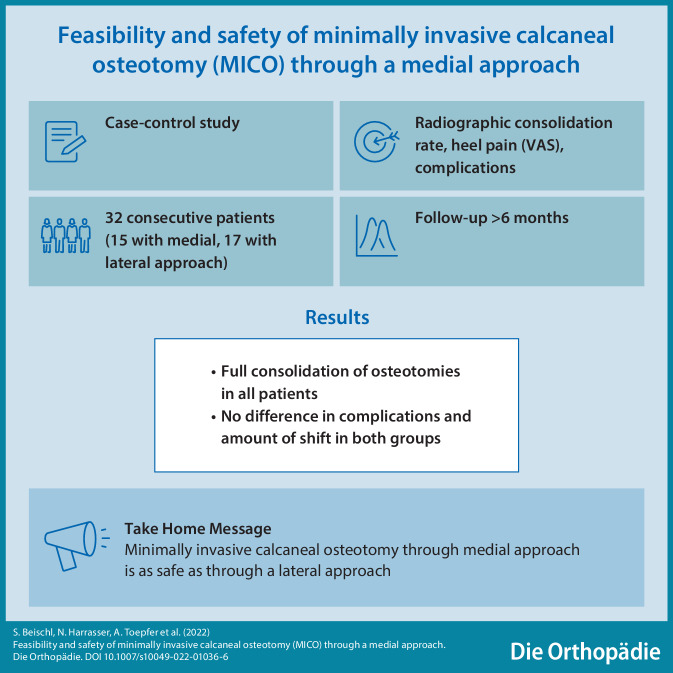

## Abstract (de)

**Einleitung:**

Die minimal-invasive Kalkaneusosteotomie (MICO) ist bereits ein etabliertes chirurgisches Verfahren zur Korrektur von Rückfußdeformitäten über einen lateralen Zugang. Ein medialer Zugang für die MICO ist bisher nicht beschrieben worden.

**Material und Methoden:**

Zwischen August 2022 und März 2023 wurde bei 32 konsekutiven Patienten (MICO mit medialem Zugang, MMICO: *n* = 15; MICO mit lateralem Zugang, LMICO: *n* = 17) im Rahmen einer komplexen rekonstruktiven Fuß- und Sprunggelenkoperation mit begleitenden Eingriffen eine MICO durchgeführt. Das Ausmaß der Korrektur in der axialen Ansicht des Fersenbeins und die Konsolidierungsraten wurden radiologisch ausgewertet. Die subjektive Zufriedenheit, die Steifheit des Subtalargelenks und das Schmerzniveau (numerische Ratingskala, NRS) auf Höhe der Ferse wurden klinisch beurteilt. Die letzte Follow-up-Untersuchung fand nach 6 Monaten statt.

**Ergebnisse:**

Alle Osteotomien konsolidierten innerhalb von 6 Monaten nach dem Eingriff. Die Verschiebung des Tuber calcanei betrug in beiden Gruppen im Durchschnitt 9 mm. Eine relevante Steifigkeit des Subtalargelenks wurde bei 5 MMICO- und bei 6 LMICO-Patienten festgestellt. Hinsichtlich Wundheilungsproblemen, Nervenschäden, Fersenschmerzen oder Patientenzufriedenheit wurden keine relevanten Unterschiede zwischen den Gruppen festgestellt.

**Schlussfolgerung:**

In dieser Studie wurden laterale und mediale Zugänge für die MICO untersucht. In beiden Gruppen wurden ähnliche Korrekturgrade und niedrige Komplikationsraten festgestellt. Der mediale Zugang für die MICO ist sicher und kann hinsichtlich der Patientenpositionierung und der Anordnung des C‑Bogens von Vorteil sein.

## Introduction

Gleich was the first to explain the idea of an osteotomy to change the axis of the calcaneus in 1893 [1]. Since then, several techniques regarding the exact placement of the osteotomy with different fixation devices and varying indications have been described [12, 15]. Nowadays, calcaneal osteotomy can be safely performed through minimally invasive (MI) incisions and are used for varying hindfoot pathologies as displacement of the tuber in all directions can be performed. When comparing minimally invasive calcaneal osteotomy (MICO) to traditional open approaches, the literature shows that MICO causes fewer complications, especially regarding wound healing [10, 23]. Whether an open or MI technique is used, the lateral approach is traditionally preferred for the osteotomy [9, 11, 18, 23, 25]. The patient is usually positioned in a lateral or supine decubitus position to allow access to the lateral heel [5, 18]. Depending on the surgeon’s dominant hand or the image intensifier’s position, this can sometimes be challenging as the foot has to be positioned close to the input window of the C‑arm. Additionally, if the hip is stiff and the leg cannot be rotated in the desired position, it can be a struggle to obtain a clear lateral view of the heel. To avoid these difficulties, we started using a medial incision for the calcaneal osteotomy in patients placed in the supine position during surgery and the leg could easily be externally rotated. In this setting, the calcaneus can be osteotomized under fluoroscopic guidance independent of the surgeon’s dominant hand. Therefore, the present study aimed to evaluate the radiographic results of the MICO via a medial approach and compare it to a control group of patients with an MI osteotomy through a lateral approach. Furthermore, complications should be reported. We hypothesized that neither group had any relevant differences regarding complications and radiographic outcome.

## Material and methods

This retrospective study includes 32 consecutive patients (MICO with medial approach, MMICO: *n* = 15; MICO with lateral approach, LMICO: *n* = 17) who underwent MICO between September 2022 and March 2023 at 3 institutions (Klinikum rechts der Isar, ATOS Klinik and Schön Klinik MHA). The study was conducted as a proof-of-principle in accordance with the Declaration of Helsinki and was approved by the local ethics review board (Technische Universität München, 2022-503-S-KH). Exclusion criteria were lost to follow-up and a patient age < 18 years. Demographic data of included patients are given in Table 1. All MICOs were part of complex reconstructive foot and ankle surgeries with concomitant procedures (Table 2). Preoperatively and 6 months postoperatively, various clinical and radiographic data were collected. Clinically, patients were asked if they would opt for the surgery again (answers: yes/unsure/no). Mobility of the operated and nonoperated subtalar joint was documented and the difference was calculated. This was determined semi-quantitatively: difference < 10°: no significant limitation, > 10°: significant limitation. Additionally, the pain level regarding heel pain (numeric rating scale NRS, 0 no pain–10 worst possible pain) was recorded postoperatively. This modification was applied because multiple other procedures that could interfere with general foot pain were necessary in these patients. Complications (nerve damage, wound healing) were documented. Radiographically, images were taken in a standardized fashion (foot under full weight bearing in dorsoplantar and lateral views, Harris view of the calcaneus), and translation of the tuber was measured. Additionally, a possible loss of correction and the osseous consolidation of the osteotomy were documented. The last follow-up was 6 months on average (5–8 months).

**Table 1:** Patient characteristics of the two groups: minimally invasive calcaneal osteotomy (MICO) with medial approach (MMICO) and MICO with lateral approach (LMICO)

	MMICO	LMICO	*p* value
Patients/feet	15/15	17/17	0.911
Age, years, mean	54.8	41.6	0.058
Gender, female/male	10/5	4/13	0.013
Side, left/right	6/9	7/10	0.303
Shift medial/lateral	11/4	12/5	0.884
No. of additional procedures, mean (range)	2 (1–5)	2 (1–5)	1.0

**Table 2:** Additional procedures performed in the medial minimally invasive calcaneal osteotomy (MMICO) and lateral MICO (LMICO) groups

	MMICO	LMICO
FDL transfer/spring ligament reconstruction	6	5
Cotton osteotomy	3	6
Hintermann osteotomy	2	5
Peroneal tendon transfer	5	4
Elevation of first metatarsal	2	3
Others	15	7

*FDL* flexor digitorum longus

### Surgical technique of the MMICO

**Fig. 1:**
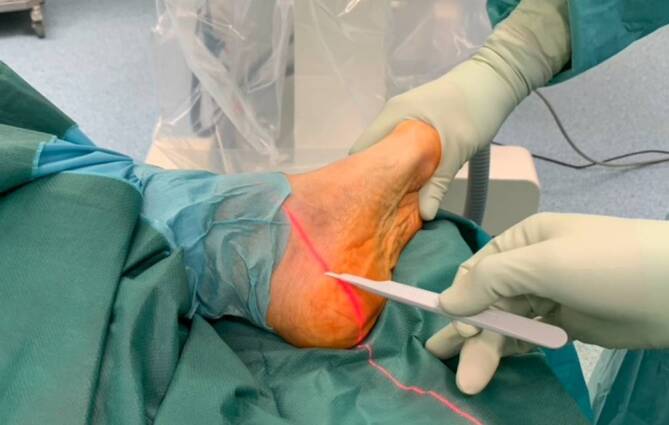
Determination of the osteotomy site with help of fluoroscopy with the leg in external rotation

**Fig. 2:**
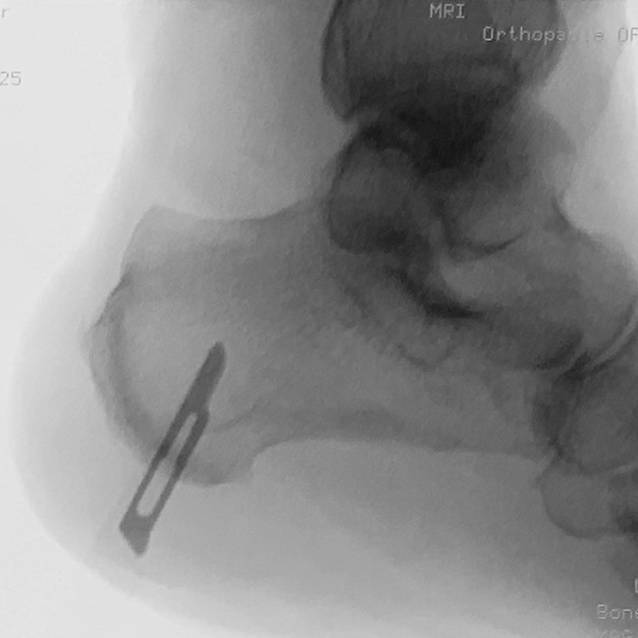
Lateral view of the fluoroscopic control of the determination of the incision

**Fig. 3:**
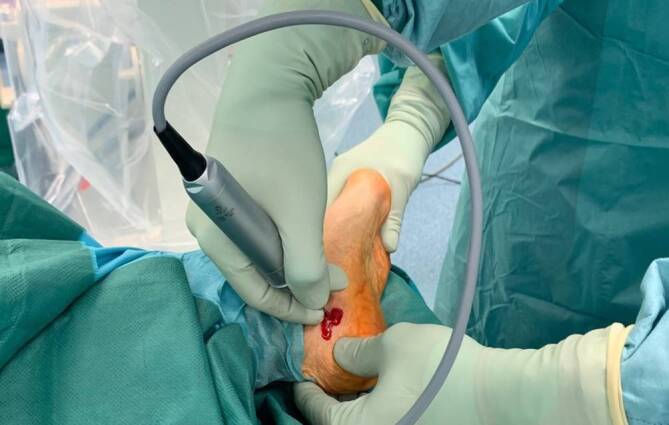
V‑shaped osteotomy through a medial incision with extrusion of bone debris

**Fig. 4:**
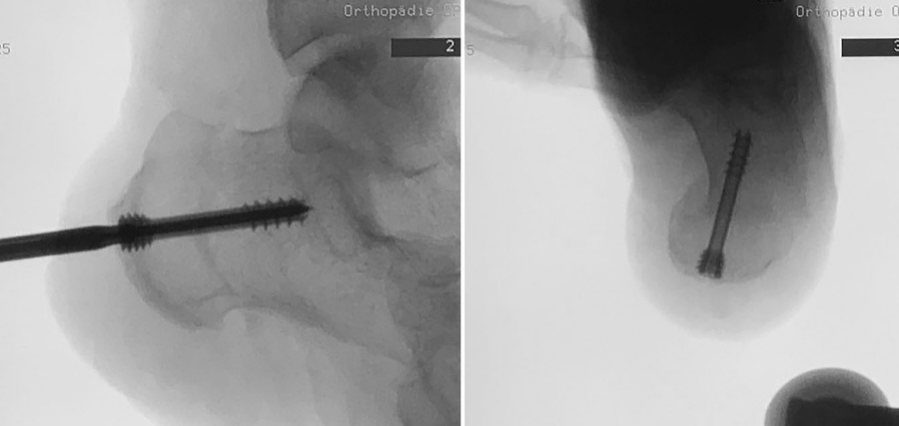
Final fluoroscopic lateral (left) and axial (right) images after insertion of one headless compression screw (6.5 mm)

All operations were performed by two fellowship-trained foot and ankle surgeons with more than 5 years of experience with MI techniques (NH performed all MMICO and 7 LMICO, HH performed 10 LMICO). The MICO was performed first or later during foot reconstruction depending on the concomitant procedures. For the lateral approach, the patient was positioned on the side with the operated foot up or in a supine position with an internally rotated leg. For the MMICO, the patient was supine with the operated leg in 90° external rotation to allow a clear lateral view of the calcaneus. A tourniquet was always applied but not inflated if the MICO was performed first; if the MICO was performed later, the tourniquet was inflated and not routinely opened for the MICO. The osteotomy cuts were V‑shaped if no cranial or caudal translation was desired. Under fluoroscopic guidance, a short incision was centered in the safe zone described by Talusan et al. ([21]; Figs. 1 and 2). A small hemostat and a periosteal elevator were used to remove the soft tissues from the bone. The 2 × 20 mm Shannon burr was inserted, and the osteotomy was performed (Fig. 3). During cutting, the burr was rotated with 6000–8000 revolutions per minute under permanent irrigation with saline. After completion of the osteotomy, the tuber was shifted in the desired direction with the use of an elevator that was introduced through the incision. The tuber was fixed with two K‑wires inserted from posterior. Following a fluoroscopic control via axial and lateral views of the calcaneus, the K‑wires were replaced with one or two cannulated screws (Fig. 4).

### Statistical analysis

Normal distribution was verified using D’Agostino-Pearson testing. An independent t‑test and a Mann-Whitney U‑test were performed for normal and non-normal distributed data to describe significant differences between the groups. Statistical significance was assumed for all *p*-values < 0.05 [23]. All analyses were performed with Python 3.9.6 (https://www.python.org/) and the scipy-library (https://scipy.org/).

## Results

### Clinical results

Overall, we found comparable satisfaction rates regarding surgical outcomes in both groups. Pain levels (NRS) at the heel were comparable between the groups (MMICO: 10 patients 0, 4 patients 1, 1 patient 2; LMICO: 8 patients 0, 7 patients 1, 2 patients 2). Regarding patient satisfaction 12 of 15 patients after MMICO (80%) and 12 of 17 patients after LMICO (70%) would opt for the surgery again. The rest were unsure but no patient would not opt for the surgery again. Complications are given in Table 3. We found delayed wound healing in one case in both groups. In both cases, the wound healed with conservative measures within 4 weeks of surgery. There was temporary dysesthesia in 4 cases (2 in each group), no relevant nerve damage persisted over 6 weeks. Regarding significant subtalar joint stiffness, we found five patients in the MMICO and six patients in the LMICO group.

**Table 3:** Summary of complications after MMICO and LMICO

	MMICO (*n* = 15)	LMICO (*n* = 17)	P value
Delayed wound healing	1 (6.7%)	1 (5.9%)	0.93
Stiffness subtalar joint	5 (33.3%)	6 (35.3%)	0.91
Nerve damage (persistent)	0 (0.0%)	0 (0.0%)	1.0

*MMICO* medial minimally invasive calcaneal osteotomy, *LMICO* lateral minimally invasive calcaneal osteotomy

### Radiographic results

All osteotomies achieved radiological consolidation within 6 months of surgery, and no signs of loosened screws were documented. The displacement of the tuber was, on average 9.3 mm (range 5.7–11 mm) in MMICO, and 9.1 mm (range 6.3–12 mm) in LMICO.

## Discussion

The main findings of the present study are that no differences regarding the amount of displacement of the tuber and risk of nerve damage were found, independent of whether a medial or lateral incision for the MICO was chosen. Moreover, complete consolidation of all osteotomies was achieved within 6 months of surgery. The safety of the medial incision, in conjunction with its simplicity regarding placement of the foot and C‑arm offers some advantages in the setting of complex reconstructive procedures in which the MICO usually represents only a small step [3, 17–19].

**Fig. 5:**
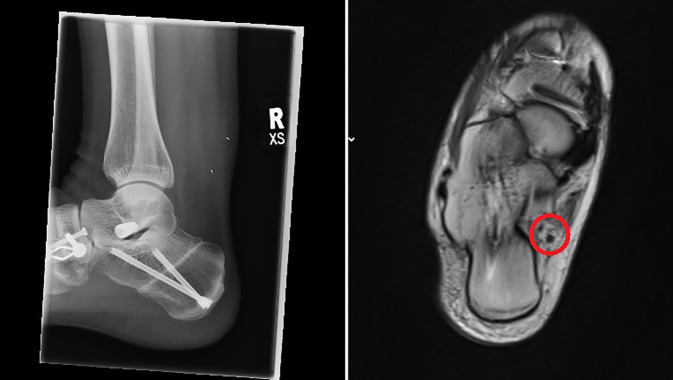
X‑ray lateral (*left*) and MRI axial images (*right*) after flatfoot reconstruction. Even though the calcaneal osteotomy does not seem to be excessively too distal, the neurovascular bundle medially is at risk (*red circle*)

Minimally invasive calcaneal osteotomies offer the same excellent clinical results and mechanical correction with a lower complication rate as open techniques [9, 11]. Therefore, these techniques are gaining more and more acceptance among foot surgeons, and multiple studies confirm these findings (Table 4). Traditionally, calcaneal slide osteotomies are performed through a lateral approach. Here, the medial neurovascular bundle is not at direct risk; therefore, this approach is safe. Nevertheless, the neurovascular bundle can be at risk even if a lateral approach is used and the cut is too distal (Fig. 5). In this context, a safe zone of the skin incision and osteotomy cut have been defined [21]. To our knowledge, no medial incision for a MICO has been described. In our patients treated with MMICO, we could not observe any permanent damage to the neurovascular bundle medially or cutaneous nerves laterally, suggesting this procedure is safe. One might suppose that a medial incision places the medial structures at risk of damage due to its anatomical proximity. We believe the contrary occurs. After medially incising the skin, the soft tissue containing nerves can easily be protected with the nick-and-spread technique. Once the burr is inserted into the bone (safe hole technique), the medial structures cannot be damaged. Another advantage of the medial approach is that if the leg is externally rotated by 90°, the C‑arm can be entered from both sides and positioned independently of either the surgeon’s preferred hand or the patient’s operated foot side.

**Table 4:** MICO studies published so far with main findings

Study	Year	Patients	Follow-up	Result
Waizy et al. [4]	2018	*60 patients*	Minimum 12 weeks	Significantly shorter operating time (MICO)
		33 open		A shorter incision in the MICO group
		27 MICO		
Kendal et al. [5]	2015	*81 patients*	30 days postoperative (primary outcome-measure)	Calcaneal displacement 9.4 mm (MICO) and 10.2 mm (open)
		50 open		Significantly fewer wound complications (MICO = 6.45% vs. open = 28%)
		31 MICO		Significantly lower rate of wound infection (MICO = 3% vs. open = 20%)
				3 patients in the open group experienced sural peripheral neuropathy
				Nonunion occurred in 1 patient (MICO)
Gutteck et al. [24]	2019	122 patients:	1‑year postoperative	Lower rate wound complications (MICO) and 15.5% (open)
		58 open		No nonunions in both groups
		64/66 patients/feet MICO		Sural nerve damage 4 (MICO) and 8 patients (open)
				Hardware removal because of discomfort 4 (MICO) and 5 (open) patients
				Revision 0 (MICO) and 6 (open)
				No significant differences in radiological outcome
				Average length of stay was 4 (MICO) and 7 (open) days *p* < 0.0001
Kheir et al. [9]	2015	29 MICO patients	Minimum 6 weeks	No wound complications
				Radiological and clinical union occurred in all cases (100%)
				No neurovascular injuries
Jowett et al. [7]	2015	9 MICO cadaveric specimens	Minimum 6 months	Uninjured neurological structures in all 9 specimens
		35 MICO patients		Radiological and clinical union occurred in all 35 patients (100%)
				No neurovascular complications
				No wound complications

*MICO* minimally invasive calcaneal osteotomy

In our study, 28.1% of osteotomies were lateralizing. It is described that this shift can be somewhat more troublesome because the medial nerves are set under compression. To avoid this complication, some authors recommend protective tarsal tunnel release when performing lateralizing calcaneal osteotomies [2, 13, 24], especially when lateralization of the calcaneal tuberosity of more than 8 mm is performed [6]. Other authors state no beneficial effect with the tarsal tunnel release [20, 22].

One study showed that a lateralizing calcaneal osteotomy performed via an open medial approach had a clinically negligible incidence of neurologic injury while adequate translation was achieved to obtain correction of varus hindfoot deformity. The authors believed there is a less direct and less percussive injury to branches of the tibial nerve when performing the osteotomy from medial to lateral [8]. In our study, we could not find any signs of permanent paresthesia at the level of medial or lateral plantar nerves suggestive of nerve compression in the tarsal tunnel. This can be explained by the fact that we did not perform the MICO on patients with neurological diseases such as hereditary motor sensory neuropathy (HMSN) as, in these patients, the risk of tarsal tunnel syndrome in conjunction with lateral sliding calcaneal osteotomies is thought to be higher [4, 13, 14, 16]. Additionally, we performed a lateral sliding of the tuber not exceeding 15 mm. Furthermore, we agree with other authors that if the medial approach is used the periosteum of the medial calcaneus is routinely elevated or perforated with the Freer before the burr is entered into the bone [8]. This release might also put less stress on the tarsal tunnel if the tuber is slid.

Our study has some limitations. First, it is a retrospective case series with a relatively small sample size, although the number of patients is comparable to other studies dealing with MICO through a lateral approach [9, 11]. Second, the follow-up of 6 months is short. Nevertheless, after 6 months, all osteotomies were consolidated; therefore, the study’s main question regarding the safety of the MMICO could be answered. Third, we did not use a clinically established patient-reported outcome measure (PROM) to evaluate clinical satisfaction after MICO. Therefore, we were not able to objectively compare the results of both groups. On the other hand, this is not possible in our study as MICO was only one part of other procedures (Table 2), and therefore, it cannot be stated which amount of the whole clinical outcome is the result of the MICO. Additionally, although PROMs may be valuable in comparison of various surgical treatments and differences between distinct population groups, clinical interpretation of these differences can sometimes be misleading [7]. Nonetheless, these limitations must be considered before conclusions about daily practical actions are drawn.

## Conclusion

This study proves that MICO through a medial incision is as safe and powerful as through a lateral incision. The medial incision does not need the lateral decubitus position, and the C‑arm can be entered from both sides without disturbing the operating field. This allows the surgeon to perform the MICO without changing the patient’s supine position in the setting of complex reconstructive procedures of the foot.

## Abbreviations

MICO: minimally invasive calcaneal osteotomy
MMICO: medial incision for MICO
LMICO: lateral incision for MICO
MI: minimally invasive
